# Strabismus in an Adolescent With Stargardt Disease: An Atypical Presentation

**DOI:** 10.7759/cureus.69325

**Published:** 2024-09-13

**Authors:** José J López-Fontanet, Gabriel Guardiola Dávila, Natalio Izquierdo, Armando Oliver

**Affiliations:** 1 Department of Ophthalmology, School of Medicine, Medical Sciences Campus, University of Puerto Rico, San Juan, PRI; 2 Department of Surgery, School of Medicine, Medical Sciences Campus, University of Puerto Rico, San Juan, PRI

**Keywords:** abca4 gene, exotropia, ocular disease, retina, stargardt disease

## Abstract

We report on the case of a 19-year-old male with Stargardt disease (STGD1) who presented with a five-year history of progressive vision loss, accompanied by the recent onset of alternating exotropia. This patient initially sought care due to difficulties with near vision and tended to focus on distant objects when looking to the right. He was found to have a best-corrected visual acuity of 20/200 in both eyes. A comprehensive evaluation, including multimodal imaging and multifocal electroretinogram, was performed. Genetic testing confirmed the diagnosis of STGD1, identifying homozygous mutations in the ABCA4 gene. Interestingly, an additional heterozygous mutation in the WDPCP gene, typically associated with Bardet-Biedl syndrome, was also discovered. The patient's exotropia, an atypical feature in STGD1, underscores the importance of comprehensive clinical and genetic evaluation and the need for further research into the clinical significance of these findings.

## Introduction

Stargardt disease (STGD1) is an inherited macular dystrophy characterized by progressive vision loss [1]. The incidence of STGD1 is approximately 10 to 12.5 per 100,000 people [2]. Ocular manifestations of STGD1 include progressive bilateral central vision loss, which most frequently begins during childhood with a secondary peak in early adulthood, associated with peripapillary atrophy, macular atrophy, and pisciform flecks [3-6].

STGD1 is typically inherited as an autosomal recessive trait. Mutations in the ABCA4 gene remain the most common cause [7]. The ABCA4 gene encodes a protein part of the adenosine triphosphate (ATP)-binding cassette family, found in rod and cone photoreceptors [8]. Mutations in the ABCA4 gene lead to the accumulation of toxic byproducts in the retinal pigment epithelium (RPE), causing degeneration of photoreceptors and RPE cells [3-6]. Diagnosis is primarily based on clinical phenotypic findings; however, genetic testing may confirm the genotypic diagnosis and identify the specific mutations involved in each patient. Although no established therapy exists for patients with STGD1, emerging treatments such as gene therapy and stem cell therapy are currently under investigation [6]. Photoprotection and avoiding vitamin A supplementation are currently recommended to prevent toxic byproduct formation in the retinal pigment epithelium and, thus, slow disease progression [6].

We report on a 19-year-old male patient with bilateral low vision and alternating exotropia and the characteristic retinal features of STGD1, including peripapillary atrophy, macular atrophy, and pisciform flecks.

## Case presentation

A 19-year-old male patient was referred to our clinic by his optometrist. The patient complained of a slowly progressive loss of vision over the last five years, worst at near distances. Additionally, over the past two months, his family observed that his right eye deviated, and he tended to focus on distant objects when looking to the right. The patient denied experiencing flashes, photophobia, or nyctalopia. There was no family history of eye diseases or past ocular diseases. A review of systems, toxic habits, and social history was unremarkable.

Upon a comprehensive ophthalmic evaluation, his best corrected visual acuity was 20/200 in both eyes (OU). The cycloplegic refraction was -2.00 +1.50 x 90 in the right eye (OD) and -1.50 +1.50 x 90 in the left eye (OS). Extraocular movements revealed alternating exotropia, with measurements showing 16 prism diopters by the Krimsky test in all gazes OU. Ductions and versions were full and unrestricted in both eyes. The cover-uncover test demonstrated alternating exotropia, and the strabismus was found to be concomitant, with no significant variation in deviation across different fields of gaze. An Ishihara color-plate test was used to assess the patient’s color vision and revealed no defect in either eye. Upon a Goldmann applanation tonometry, intraocular pressure was 12 mmHg and 13 mm Hg OD and OS, respectively. The patient had normal adnexa and an anterior segment slit lamp examination OU. Pupils were reactive without an afferent pupillary defect OU.

**Figure 1:**
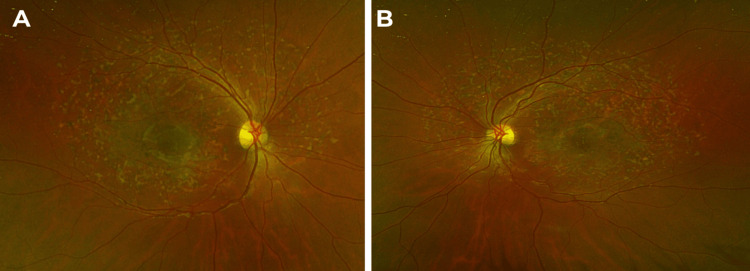
Wide-field fundus photographs OD (A) and OS (B) show mild temporal optic nerve pallor, peripapillary atrophy, and mottling with pigment deposition in the macula. Pisiform flecks are present throughout the macula and periphery, sparing the peripapillary retina. OD, right eye; OS: left eye.

As depicted in Figure 1, fundus examination showed mild temporal optic nerve pallor, peripapillary atrophy, pigment deposition, and mottling in the macula, fundus pisiform flecks sparing the peripapillary retina OU.

**Figure 2:**
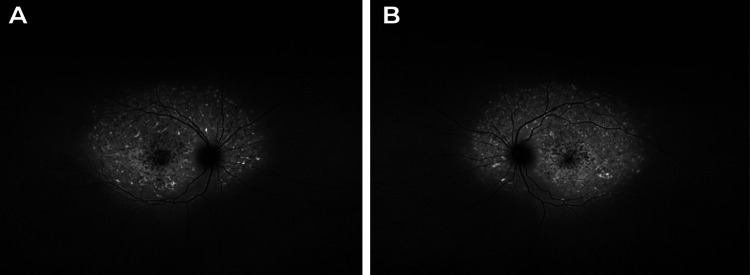
FAF images OD (A) and OS (B) demonstrate a central area of decreased autofluorescence, encircled by a ring of increased autofluorescence and hyper-autofluorescent pisciform flecks in OU. OD, right eye; OS: left eye; OU, both eyes.

Fundus autofluorescence (FAF) images revealed a central area of decreased autofluorescence, encircled by a ring of increased autofluorescence and hyper-autofluorescent pisciform flecks OU, as shown in Figure 2.

**Figure 3:**
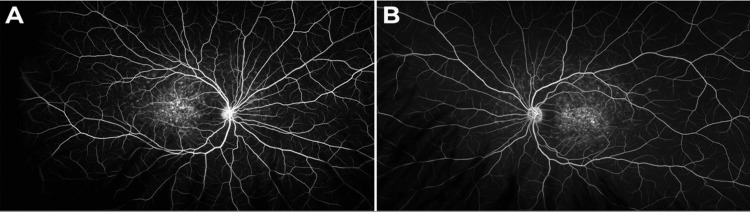
Fluorescein angiogram images of the OD (A) and OS (B) reveal central and perimacular transit defects, corresponding to macular atrophy and pisciform flecks, respectively. Additionally, a dark choroidal flow is evident on the angiogram. OD, right eye; OS: left eye.

A fluorescein angiogram revealed central and perimacular transit defects, corresponding to macular atrophy and pisciform flecks, as well as a dark choroidal flow in OU, as shown in Figure 3.

**Figure 4:**
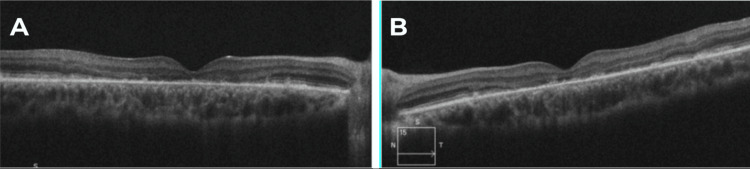
Right (A) and left (B) spectral domain OCT images reveal loss of the central ellipsoid zone and hyperreflective deposits in OU. OCT, optical coherence tomography; OU, both eyes.

A spectral-domain macular optical coherence tomography (OCT) showed loss of the central ellipsoid zone and hyperreflective deposits at the level of the RPE and the photoreceptor outer segments in OU, as shown in Figure 4. Multifocal electroretinography results revealed significant functional losses in OU.

Next-generation sequencing (NGS) and deletion/duplication analysis done by Invitae Corporation (San Francisco, CA, USA) showed homozygous mutations in the ABCA4 gene. The variant was c.52C>T (p.Arg18Trp). Interestingly, he had an additional heterozygous mutation in the WDPCP gene of the variant c.979C>T (p.GIn327*).

## Discussion

Previous studies described patients with STGD1 with ocular saccades [9], but to our knowledge, there have been no reports of patients with STGD1 presenting with strabismus. Our patient exhibited alternating exotropia measuring 16 prism diopters by the Krimsky test in all gazes OU, an unusual finding in STGD1 patients. This was one of the initial complaints that prompted the patient to seek medical evaluation. This atypical presentation underscores the need for further investigation.

Imaging findings in our patient align with those commonly reported in STGD1, demonstrating advanced and symmetric disease progression [10]. The fundus examination, FAF, fluorescein angiography, and spectral-domain OCT collectively revealed characteristic features, including optic nerve changes, macular abnormalities, and significant functional impairment confirmed by multifocal electroretinography.

Genetic testing confirmed the diagnosis, with homozygous mutations in the ABCA4 gene, which is consistent with the autosomal recessive inheritance pattern of STGD1. Interestingly, our patient also had a heterozygous mutation in the WDPCP gene, associated with autosomal recessive Bardet-Biedl syndrome. WDPCP encodes a cytoplasmic protein predominantly found in tissues rich in ciliated cells [11]. Although this patient did not present any phenotypic features of Bardet-Biedl syndrome, the significance of this additional mutation remains unclear. Nevertheless, it highlights the complexity of genetic interactions and underscores the importance of comprehensive genetic counseling for prognosis and management.

In patients presenting with new-onset strabismus, it is crucial not to overlook a thorough fundus examination. This case highlights the importance of such an examination, as it can reveal underlying retinal diseases that may be the root cause of the motility disorder. In this patient, the presence of STGD1, a retinal condition characterized by progressive macular degeneration, likely contributed to the development of decompensated exophoria now manifesting as alternating exotropia. Retinal diseases like STGD1 can lead to exotropia through several mechanisms, including reduced central visual acuity, disruption of binocular vision, and impaired visual fixation [12-14]. These factors may cause the eye to drift outward due to the loss of foveal function, leading to a secondary ocular misalignment [12-14]. Therefore, a comprehensive retinal evaluation should be considered in patients with strabismus of uncertain origin to identify any potential underlying retinal pathology that could be contributing to the ocular misalignment.

## Conclusions

Stargardt disease (STGD1), an inherited macular dystrophy caused by mutations in the ABCA4 gene, typically presents with gradual vision loss. However, this case highlights atypical features, such as exotropia, which may be among the initial symptoms, prompting earlier medical evaluation. These findings underscore the importance of routine ocular examination and comprehensive genotypic studies, which can reveal additional mutations of uncertain significance. Understanding these atypical presentations is crucial for timely diagnosis and intervention. Further research is needed to elucidate the mechanisms linking retinal dysfunction to motility disorders and to develop targeted treatments that improve patient outcomes in STGD1. Given the progressive nature of STGD1, the prognosis for this patient includes ongoing visual decline, and while no cure currently exists, protective measures such as UV-blocking eyewear, avoidance of vitamin A supplementation, and regular monitoring may help slow the pace of vision loss.
